# Cardamonin induces G2/M phase arrest and apoptosis through inhibition of NF-κB and mTOR pathways in ovarian cancer

**DOI:** 10.18632/aging.104184

**Published:** 2020-11-25

**Authors:** Jiang Ruibin, Jin Bo, Wan Danying, Feng Jianguo, Gu Linhui

**Affiliations:** 1 The Cancer Hospital of the University of Chinese Academy of Sciences (Zhejiang Cancer Hospital), Institute of Basic Medicine and Cancer (IBMC), Chinese Academy of Sciences, Hangzhou 310022, Zhejiang, China; 2 College of Life Science, Zhejiang Chinese Medical University, Hangzhou 310053, Zhejiang, China

**Keywords:** cardamonin, apoptosis, NF-κB, mTOR, ovarian cancer

## Abstract

Cardamonin, a natural chalcone, is reported to induce apoptosis and inhibit cancer cell growth. However, the mechanisms underlying the therapeutic effects of cardamonin remain to be established. Here, we have focused on cardamonin-induced apoptosis in ovarian cancer cells, both *in vitro* and *in vivo*. The effects of cardamonin on cell cycle patterns and apoptotic responses of cells were assessed in this study. Western blot was employed to determine the effects of cardamonin on expression of cell cycle- and apoptosis-related proteins. Our results indicate that cardamonin suppresses cancer cell growth by inducing G2/M phase arrest and apoptosis through targeted inhibition of NF-κB and mTOR pathways. The collective findings provide novel insights into the pathways responsible for the anticancer effects of cardamonin and support its potential utility as a clinical therapeutic agent for ovarian cancer.

## INTRODUCTION

Ovarian cancer is the most common tumor type in women worldwide and a leading cause of mortality, with more than 204,000 cases diagnosed annually. Based on cancer stage, the first line of treatment is surgery, followed by radiation, hormone therapy and chemotherapy. In view of the common occurrences of metastasis and disease resistances to chemodrugs and radiotherapy, however, the development of effective therapeutic agents for ovarian cancer remains an urgent unmet clinical need [1–3].

Members of the nuclear factor κB (NF-κB) family of proteins act as transcription factors promoting the expression of multiple genes [4–6]. Recently, constitutive expression of NF-κB has been associated with several cancer types [7, 8]. In addition to direct effects on cancer cells, NF-κB may also impact immune cells to prevent tumor development [9]. In the canonical pathway, NF-κB dimers are regulated by inhibitory molecules of the IκB family, which prevent their translocation into the nucleus through interacting with each other and forming a stable complex. To promote release of the NF-κB complex, signaling pathways are activated by pro-inflammatory cytokine receptors. The receptors activate the IκB kinase (IKK) complex (IKKα, IKKβ and IKKγ (NF-κB essential modulator)), which phosphorylates IκB and thus facilitates its ubiquitination [10–12].

The mammalian target of rapamycin (mTOR) is closely associated with tumorigenesis and ovarian cancer development [13–15] and its downregulation inhibits cancer cell proliferation and metastasis [16–18]. mTOR is considered an important biomarker, with recently developed mTOR inhibition strategies having achieved significant success in cancer treatment [19–21]. Raptor and PRAS40 are the specific components of mTORC1. Raptor mediates translocation of mTOR to lysosomes, an essential step for mTOR activation. Additionally, PRAS40 binds Raptor and participates in the regulation of mTOR activation and cell proliferation [22, 23]. To date, several studies have shown that chalcone compounds inhibit mTORC1 signaling through disrupting the associations among mTOR, PRAS40 and Raptor [24, 25]. Interestingly, PRAS40 was recently reported to associate with p65 and modulate NF-κB transcription activity [26]. Therefore, the identification of novel agents that target and intervene with NF-κB and mTOR signaling should aid in effective treatment of ovarian cancer and improvement of clinical outcomes.

Cardamonin, a chalcone component of *Alpinia Katsumadai* with anti-tumor [27], anti-inflammatory [28] and anti-nociceptive [29] activities, has been shown to enhance the therapeutic index of cisplatin [30]. Cardamonin acts as a chemopreventive agent in multiple cancer types, including breast, lung, prostate and nasopharyngeal cancer. The compound promotes apoptosis in ovarian cancer cells [31], which is proposed to contribute to its activity in inducing cancer cell death. However, the precise anti-tumor mechanisms of action remain poorly understood.

In this study, we focused on the mechanisms underlying cardamonin-induced inhibition of ovarian cancer cell growth, both *in vivo* and *in vitro*. Mechanistically, cardamonin promoted apoptosis and G2/M phase arrest in SKOV3 cells. Furthermore, cardamonin-induced cell apoptosis was mediated through inhibition of the NF-κB and mTOR pathways. Taken together, our data support the clinical suitability of cardamonin as a candidate drug for ovarian cancer.

## RESULTS

### Antitumor effects of cardamonin on ovarian cancer cells

**Figure 1:**
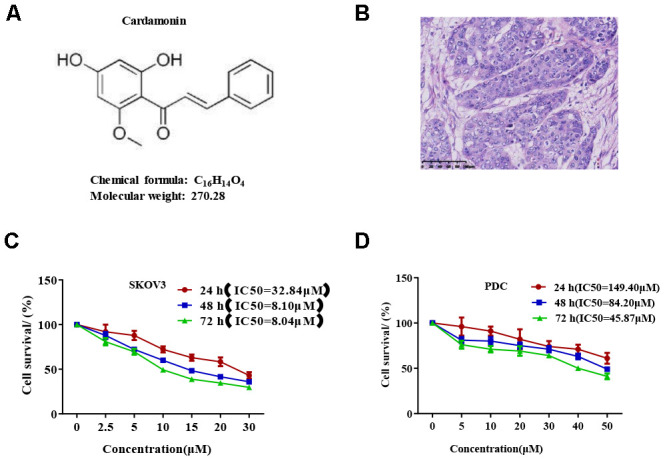
**Cardamonin inhibits SKOV3 ovarian cancer cell growth.** (**A**) Chemical structure of the active form of (E)-1-(2,4-dihydroxy-6-methoxypheny)-3-phenylprop-2-en-1-one (cardamonin). (**B**) Image showing H&E staining of original tumor dissected from patient. (**C**, **D**) Graph showing the inhibitory effects of cardamonin on proliferation of SKOV3 and PDC cells. Average results from three independent experiments are presented; SD signifies standard deviation (n=3).

**Figure 2:**
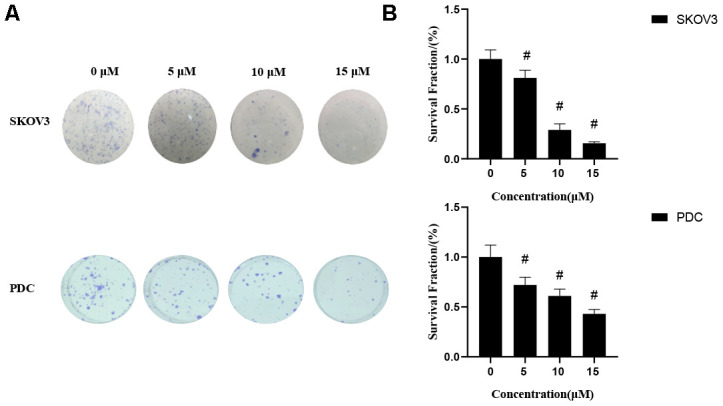
**Effects of cardamonin on clonogenic survival of SKOV3 and PDC.** (**A**) Representative images showing clonogenic survival assays with SKOV3 and PDC cells. (**B**) Graphs showing changes in the clonogenic survival fraction. SD signifies standard deviation, #*P* < 0.05 versus control group, n=3.

We first determined the inhibitory effects of cardamonin on proliferation of SKOV3 and PDC cells. In this experiment, we used DMSO as the negative control. MTT assay result showed that cardamonin inhibited proliferations of SKOV3 and PDC cells to a significant extent in a dose-dependent manner (Figure 1). The median inhibition concentration (IC_50_) values at 24 h, 48 h, and 78 h were 32.84 μM, 8.10 μM, and 8.04 μM, respectively. (c) Graph showing the inhibitory effect of cardamonin on proliferation of patient-derived cells (PDC). The median inhibition concentration (IC_50_) values at 24 h, 48 h, and 78 h were 149.40 μM, 84.20 μM, and 14.87 μM, respectively. In addition, we observed dose-dependent suppressions of colony formation in SKOV3 and PDC cells treated with cardamonin (Figure 2).

### Cardamonin causes cell cycle arrest in SKOV3 and PDC cells

**Figure 3:**
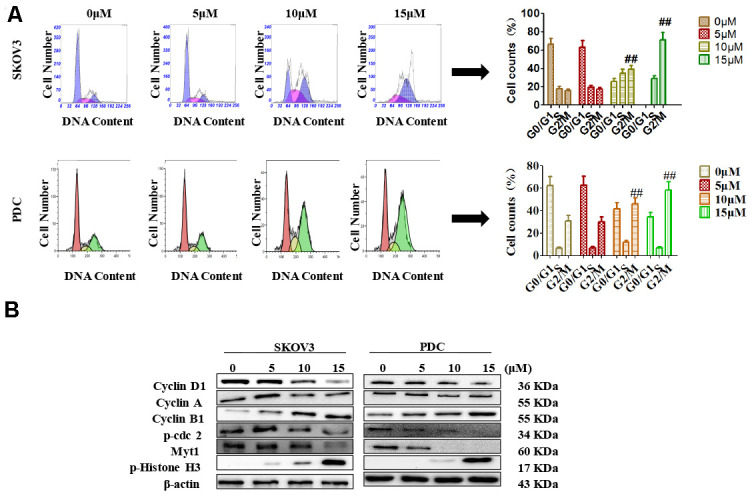
**Effects of cardamonin on cell cycle distribution of ovarian cancer cells.** (**A**) Cells were treated with different concentrations of cardamonin for 48 h. Representative flow cytometry data on SKOV3 cells and PDC treated with cardamonin. Graphs showing changes in the percentage of cells at each phase of the cell cycle for both SKOV3 and PDC groups. (**B**) Representative western blots showing the effects of cardamonin on expression of cyclin D1, cyclin A, cyclin B1, p-cdc2, Myt1 and p-Histone H3. Data represent average results from three independent experiments; SD signifies standard deviation (n=3), **P<0.01 versus control group.

Recent studies have shown that cardamonin induces cell cycle arrest through modulation of cell cycle regulators in ovarian cancer. In this study, we determined cell cycle changes of SKOV3 and PDC cells after treatment with cardamonin. As shown in Figure 3, we found that treatment with cardamonin induced a significant increase of the percentage of cells at the G2/M phase in both SKOV3 and PDC groups.

Cyclin D1, cyclin A, cyclin B1, p-cdc2, Myt1 and p-histone H3 are critical molecules in cell cycle regulation. We thus used Western blot to evaluate the changes of protein levels of these potential regulatory molecules. We found that treatment with cardamonin resulted in a concentration-dependent increase on expression of cyclin D1, cyclin B1 and p-histone H3, along with a concomitant decrease of cyclin A, p-cdc2 and Myt1 protein expressions.

### Cardamonin induces apoptosis in SKOV3 cells and PDC

**Figure 4:**
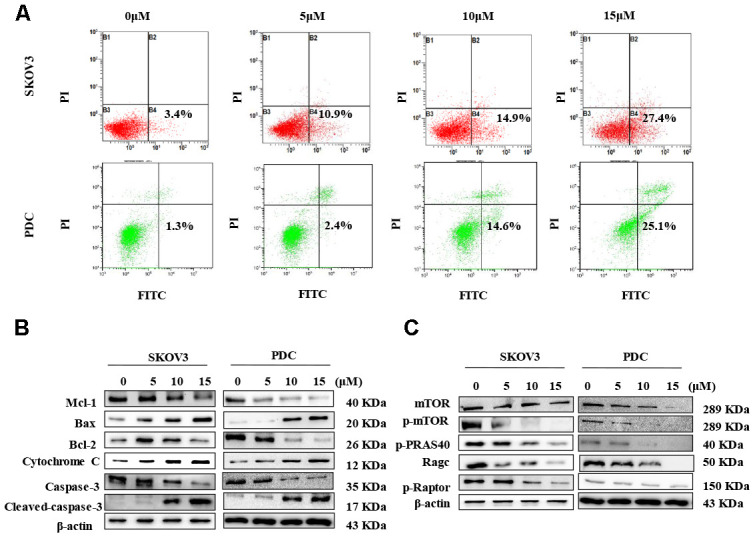
**Cardamonin treatment induces apoptosis and inhibits the mTOR pathway in ovarian cancer cells.** (**A**) Representative images of apoptosis detected with Annexin V-FITC and PI staining in SKOV3 cells and PDC. Cells were treated with different concentrations of cardamonin for 48 h. Percentages in the bottom right and top right quadrants represent early and late apoptosis, respectively. (**B**) Western blot showing change of protein expressions of Mcl-1, Bax, Bcl-2, cytochrome C, caspase-3 and cleaved caspase-3. (**C**) Western blot showing chang of protein expression of mTOR, p-mTOR, p-PRAS40, Ragc, and p-Raptor. *β*-actin was included as a loading control.

In view of data from the MTT assay showing that cardamonin induces cytotoxicity, we examined SKOV3 cells and PDC treated with cardamonin for potential evidence of apoptosis. Double staining with both Annexin V-FITC and PI was employed to distinguish apoptotic cells from other cell populations. Interestingly, we observed a significant increase in both early and late apoptosis in SKOV3 and PDC groups treated with cardamonin (Figure 4A).

Western blot analysis was further conducted to determine the pathways underlying cardamonin-induced apoptosis. Bcl-2 and the caspase cascade play essential roles in balancing anti-apoptosis and proapoptosis processes and integrate diverse upstream survival and death signals to determine cell fate. In our experiments, Mcl-1, Bcl-2 and caspase-3 levels were decreased in cells treated with cardamonin whereas levels of Bax, cytochrome C and cleaved caspase-3 were increased (Figure 4B). We also detected reduced apoptosis index in cells with co-treatment of Z-VAN-FMK (a pan-caspase inhibitor) and cardamonin (Figure 7E). These results thus suggest that cardamonin regulates expression of Bcl-2 family proteins and activates the caspase cascade to promote apoptosis in ovarian cancer cells.

### Activation of mTOR is attenuated by cardamonin

To gain further insights into the molecular mechanisms underlying the cardamonin-mediated decrease in viability, we investigated the expression and phosphorylation patterns of mTOR, p-PRAS40, p-Raptor and Rag in SKOV3 and PDC cells. Our data showed significant cardamonin-induced reduction of p-mTOR, p-PRAS40, p-Raptor and Ragc levels (Figure 4C). Since Raptor and PRAS40 are the specific components of mTORC1, we treated cells with cardamonin and a mTOR inhibitor, rapamycin. We found that rapamycin and cardamonin showed similar effects on apoptosis (Figure 7B), cell cycling (Figure 6B) and the protein expression patterns in cells (Figures 6D, 7D). Of interest, we also observed that the treatment with rapamycin diminished the effects of cardamonin on apoptosis induction and cell cycle arrest, which strongly suggested the involvement of mTOR signaling in the cardamonin acting pathway.

### Cardamonin inhibits NF-κB signaling

**Figure 5:**
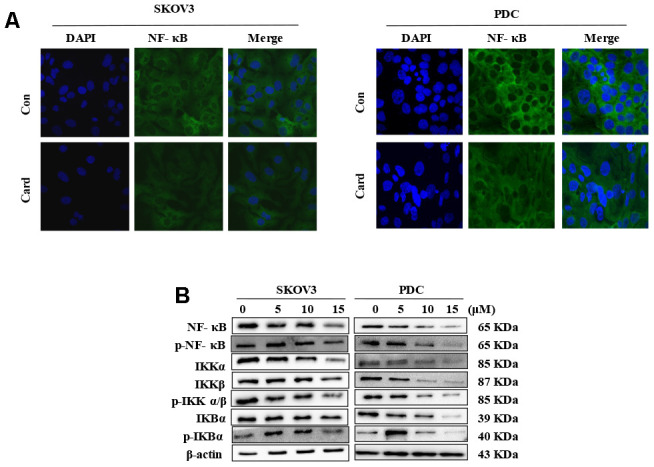
**NF-κB mediates the anti-proliferative effect of cardamonin in ovarian cancer cells.** (**A**) SKOV3 cells and PDC were cultured with 15 μM cardamonin or 0.1% DMSO for 24 h and intracellular distribution of NF-κB subsequently analyzed via immunofluorescence. (**B**) Western blot showing change of protein expressions of NF-κb, p-NF-κB, IKKα, IKKβ, p-IKKα/β, IKBα and p-IKBα in cells after treatment with the indicated concentrations of cardamonin. *β*-actin was included as a loading control.

**Figure 6:**
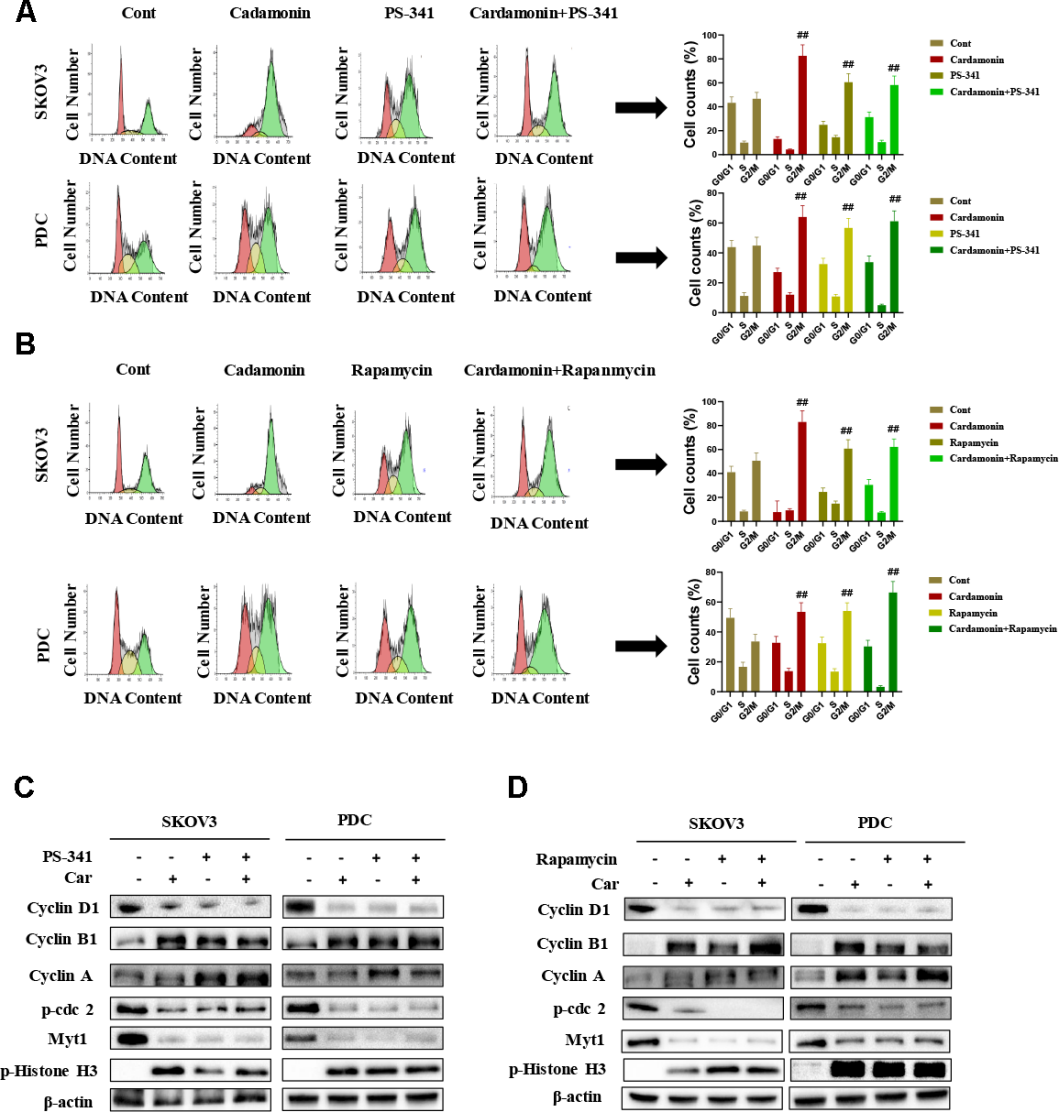
**Cardamonin induces cell cycle arrest in SKOV3 and PDC cells through NF-κB and mTOR pathways.** (**A**) Representative results of flow cytometry analysis of SKOV3 cells and PDC treated with cardamonin (15 μM) or PS-341 (50 nM). Graphs depicting changes in the percentage of cells at each phase of the cell cycle. (**B**) Representative results of flow cytometry analysis of SKOV3 and PDC treated with cardamonin (15 μM) or rapamycin (20 nM). Graphs depicting changes in the percentage of cells at each phase of the cell cycle. (**C**) Western blots showing the effects of cardamonin (15 μM) and PS-341 (50 nM) on expression of cyclin D1, cyclin A, cyclin B1, p-cdc2, Myt1 and p-Histone H3. (**D**) Western blots showing the effects of cardamonin (15 μM) and rapamycin (20 nM) on expression of cyclin D1, cyclin A, cyclin B1, p-cdc2, Myt1 and p-Histone H3. Data represent average results from three independent experiments; SD signifies standard deviation (n=3), ##*P*<0.01 indicates significance.

**Figure 7:**
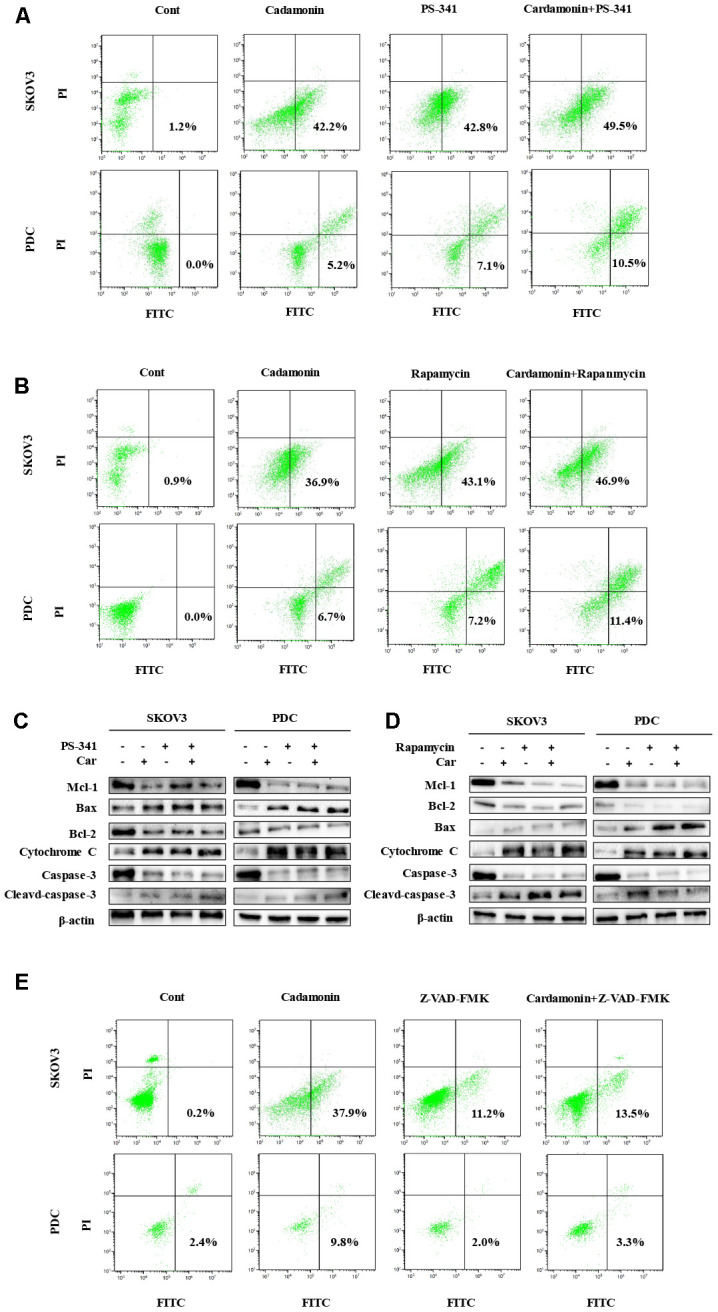
**Cardamonin induces apoptosis through NF-κB and mTOR pathways in SKOV3 and PDC.** (**A**, **B**, **E**) Representative images showing apoptosis detected with Annexin V-FITC and PI staining in SKOV3 and PDC after treatment of cardamonin (15 μM), PS-341 (50 nM) or Z-VAD-FMK(20 μM). The percentages in the bottom right quadrant and top right quadrant represent early and late apoptosis, respectively. (**C**, **D**) Western blot showing change of protein expression of Mcl-1, Bax, Bcl-2, cytochrome C, caspase-3 and cleaved caspase-3. *β*-actin was included as a loading control.

The NF-κB pathway is associated with several cancer types. Accordingly, we examined effects on the NF-κB pathway in cardamonin-treated SKOV3 and PDC groups. Interestingly, significant reduction of NF-κB protein in SKOV3 and PDC groups treated with cardamonin for 24 h was observed (Figure 5A). We additionally investigated the expression patterns of NF-κB pathway-related proteins. Treatment with cardamonin induced a marked decline in p-NF-κB, IKKα and IKKβ levels, indicative of inhibition of the NF-κB pathway. Phosphorylation of ΚBα, IKKα and IKKβ was additionally detectable (Figure 5B). To validate the influence of the NF-κB pathway on cardamonin-induced G2/M phase arrest and apoptosis, cell cycle and apoptosis were examined in the presence of PS-341 (a NF-κB inhibitor). As shown in Figures 6A and 7A, treatment with PS-341 also triggered G2/M phase arrest and apoptosis in SKOV3 and PDC cells. As expected, we detected reduced effects of cardamonin treatment on apoptosis, cell cycling and protein expressions of investigated proteins in SKOVs and PDC cells when cells were exposed to PS-341 (Figures 6C and 7C). These results thus demonstrated an association of the NF-κB pathway with cardamonin activity.

### Cardamonin inhibits tumor growth *in vivo*

To establish the *in vivo* effect of cardamonin on ovarian cancer, SKOV3 and PDC **(**3×10^6^ cells**)** were injected into the flanks of BALB/c athymic nude mice. When tumor widths reached 3-5 mm, mice were regrouped and treated with different doses of cardamonin or cisplatin. Notably, both cardamonin and cisplatin exerted significant growth inhibitory effects *in vivo*. While differences in tumor weight and volume were not significant between the two groups (Figure 6A), cisplatin clearly caused body weight loss and renal damage. In contrast, no weight loss or impairment of liver and kidney function was evident in cardamonin-treated mice (Figure 6B).

**Figure 8:**
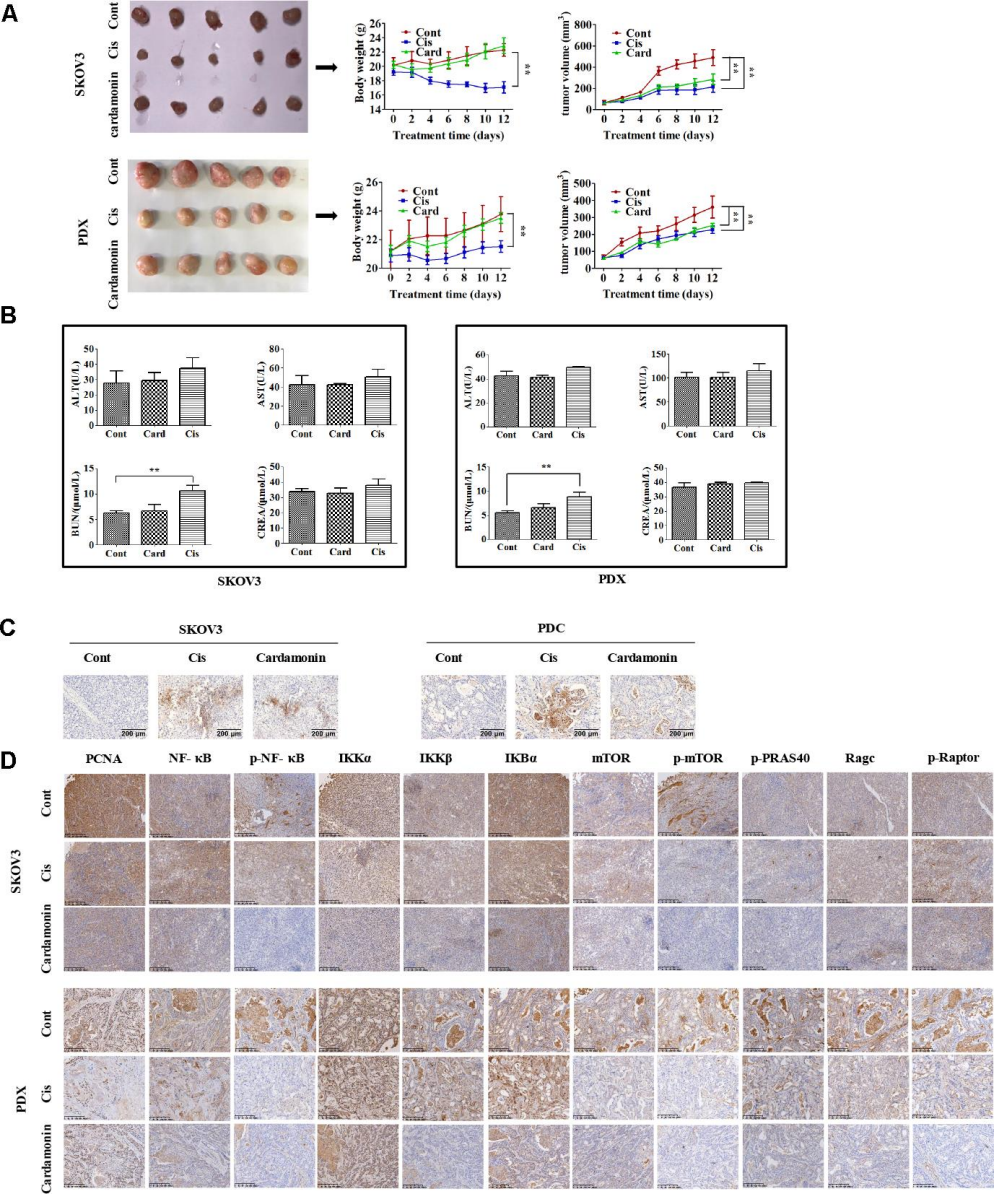
**Cardamonin inhibits tumor growth *in vivo* without serious side-effects.** (**A**) Images showing tumors collected from each group. Tumor volumes and weights were measured every other day. (**B**) Graphs showing the changes of serum levels of ALT, AST, BUN and CREA levels in mouse. (**C**) Representative images showing the apoptosis detected in xenograft tumors with TUNEL assay. (**D**) IHC results showing the staining patterns of NF-κB and mTOR pathway-related proteins in tumors, **P<0.01, n=5.

We examined apoptosis patterns in tumor sections of the xenograft tumors via the TUNEL assay. Our results showed that cardamonin could enhance apoptosis for both SKOV3 and PDC xenograft tumors (Figure 8C). IHC results further validated the modulation of NF-CB signaling and mTOR-related proteins in the xenograft tumors with cardamonin treatment.

## DISCUSSION

Data from the current study indicate that cardamonin possesses anti-tumor activity, both *in vivo* and *in vitro*. Cardamonin induces apoptosis through modulation of specific apoptotic factors and the cell cycle. Furthermore, cardamonin-mediated inhibition of ovarian cancer cell growth is achieved through suppression of mTOR and NF-κB transcriptional activity. To our knowledge, this is the first study to document cardamonin-induced G2/M phase arrest and apoptosis through inhibition of NF-κB and mTOR pathways in ovarian cancer.

Anti-tumor and anti-inflammatory effects of cardamonin have been reported. Cardamonin promotes cellular apoptosis through inhibiting NF-κB and mTOR pathways in multiple cancer types including colon cancer [32], lung cancer [33] and breast cancer [30]. However, the effects of cardamonin on ovarian cancer are yet to be established. Our present findings demonstrate that cardamonin inhibits cell proliferation in a dose-dependent manner and reduces the clonogenic survival ability of ovarian cancer cells. *In vivo*, cardamonin administered at a dose of 10 mg/kg caused a significant decrease in tumor volume, validating its antitumor activity in ovarian cancer.

Based on these results, we further examined whether apoptosis of ovarian cancer cells is induced by cardamonin. Apoptosis is a crucial process in cancer cell growth and survival. Previous studies have shown that cardamonin treatment rapidly and efficiently promotes apoptosis in different tumor types. In mitochondrial cell death signaling, accumulation of Bcl-2 family proteins and Bax/Bcl-2 ratio in the mitochondrial membrane determine the potential release of cytochrome C from the intermembrane space of mitochondria to cytoplasm, where it promotes apoptosome formation and activates the caspase cascade leading to apoptosis [34, 35]. In our experiments, cardamonin induced apoptosis in SKOV3 and PDC cells, with downregulation of Mcl-1 and Bcl-2 proteins and concomitant upregulation of Bax, cytochrome C and cleaved caspase-3 proteins. Apoptosis was detected using Annexin V-FITC and PI staining in SKOV3 and PDC cells. Our collective results suggest that mitochondria-related cell death signaling is an important contributory mechanism to cardamonin-induced apoptosis of ovarian cancer cells.

Cell cycle progression plays a critical role in cell survival. Cell cycle arrest is a prevalent step in the anti-tumor activity of numerous drugs, such as Wogonin [36], Luteolin [37], and Angustifoline [38]. Targeting of cell cycle regulators thus presents a novel strategy for cancer therapy. In view of the finding that cardamonin induces cell cycle arrest in different cancer cell types, such as breast and nasopharyngeal cancer [39], we speculated a similar function in ovarian cancer cells. Data from the current study showed a significant increase in the percentage of cells in the G2/M phase in SKOV3 and PDC groups treated with cardamonin. Cdc2 is a key regulator of cell entry into mitosis. Traversing the G2/M checkpoint to initiate mitosis requires activation of cyclin A, cyclin B and cyclin D. Phosphorylation of histone H3 is additionally essential for chromosomal segregation during mitosis. Our results indicate that G2/M arrest is an important factor underlying the anti-ovarian cancer activity of cardamonin.

NF-κB and mTOR are well-known promising therapeutic targets for cancer [40, 41]. Accordingly, we focused on whether cardamonin induces apoptosis in ovarian cancer through effects on the mTOR and NF-κB pathways. Interestingly, these pathways were inhibited in SKOV3 and PDC cells subjected to cardamonin treatment. Although this is not the first study to show that cardamonin suppresses mTOR and NF-κB signaling, to our knowledge, cardamonin-mediated reduction of mTOR and NF-κB levels in PDC as a potential anti-tumor mechanism has been demonstrated for the first time.

In conclusion, cardamonin induces G2/M phase arrest and apoptosis in ovarian cancer cells through suppression of mTOR and NF-κB signaling, supporting its potential utility as a natural therapeutic agent for ovarian cancer.

## MATERIALS AND METHODS

### Reagents

SKOV3 cells were purchased from Sigma-Aldrich Co. (St Louis, MO, USA) and cultured in McCoy's 5a medium supplemented with 10% fetal bovine serum (FBS, Gibco). Cells were screened with a Cell Culture Contamination Detection Kit (Thermo Fisher Scientific), which showed negative results for mycoplasma contamination. Cardamonin (chemical structure depicted in Figure 1A) was purchased from Aokebio (Beijing, China). PS-341, rapamycin and Z-VAN-FMK were purchased from MedChem Express (MCE, USA). Antibodies were acquired from Abcam (β-actin) and Cell Signaling Technology (cyclin D1, cyclin A, cyclin B1, p-cdc2, Myt1, p-Histone H3, Mcl-1, Bax, Bcl-2, cytochrome C, caspase-3, cleaved caspase-3, mTOR, p-mTOR, p-PRAS40, Ragc, p-Raptor, NF-κB, p-NF-κB, IKKα, IKKβ, p- IKKα/β, IKBα and p- IKBα).

Ovarian cancer tissues (Figure 1B) obtained from a female patient during surgery were used to establish a patient-derived xenograft (PDX) model and patient-derived cells (PDC). We obtained written informed consent from the patient and ethical approval from the Ethics Committee of Zhejiang Cancer Hospital (Hangzhou, China).

### Cell viability analysis

Cells were plated in 96-well plates (5×10^3^ cells/well). After 24 h, cells were exposed to increasing concentrations of cardamonin, using DMSO as a control. The MTT assay was performed for measuring cell viability according to the manufacturer's instructions (Promega, USA).

### Clonogenic assay

In total, 500 to 1000 cells were seeded into 60 mm dishes and treated with different concentrations (5 μM, 10 μM and 15 μM) of cardamonin. Cells were washed with complete medium twice after treatment for 48 h and subsequently maintained in a 37° C incubator for 10–14 days. Colonies consisting of >50 cells were considered 'surviving colonies' and directly scored using an inverted microscope after staining with crystal violet.

### Apoptosis assay

Annexin V-FITC/propidium iodide (PI) staining was performed to measure apoptotic cell death induced by cardamonin. In brief, log-phase growing cells were treated with cardamonin, harvested, and washed twice with cold PBS. Cells were resuspended in binding buffer, and stained with PI and Annexin V according to the manufacturer's instructions (BD Pharmingen, Catalog No.556547). Normal (FITC^-^/PI^-^), apoptotic (FITC^+^/PI^-^), and necrotic (FITC^+^/PI^+^) cells were quantified via flow cytometry.

### Cell cycle analysis

Cells (2.0×10^5^) were seeded in six-well plates and allowed to adhere for 24 h. Next, cells were treated with various concentrations of cardamonin for 48 h, harvested, and fixed with 70% ethanol (prepared in PBS) at -4° C. Fixed cells were washed twice with PBS, followed by incubation with 100 μg/mL RNase A and 50 μg/mL PI in PBS for 30 min in the dark. Cell cycle analysis was conducted using flow cytometry with CXP software, and the results analyzed with CytomicsTM FC 500 software (Beckman).

### Western blot analysis

Cells were collected via centrifugation, lysed in RIPA lysis buffer (Beyotime, China) containing 1 mM PMSF, and 25 μg cell lysate loaded onto 10% SDS polyacrylamide gels for immunoblot analysis. All primary antibodies were used at 1:500 to 1:1000 dilution, and beta-actin included for equal protein loading in each sample.

### Immunohistochemistry

For IHC, tumor sections were deparaffinized, rehydrated, and pretreated for 30 min in a 70° C oven. Endogenous peroxidase activity was blocked with 3% hydrogen peroxide. Heat-induced antigen retrieval was conducted for all sections in 0.01M citrate buffer, pH 6.0, at 95° C using a steamer. Sections were treated with primary antibodies diluted in PBS (1:100). After overnight incubation at 4° C, sections were incubated with Dako EnVision + System HRP Labelled Polymer for 30 min at room temperature. Sections were subsequently counterstained with hematoxylin, dehydrated, coverslipped and visualized.

### Animal studies

BALB/c nude mice (5 weeks of age, 16-18 g) were purchased from SLAC (Shanghai, China). All experiments were carried out according to the protocols of the National Institutes of Health Guide for Care and Use of Laboratory Animals. PBS (200 μL) containing 3×10^6^ cells was injected into the flanks of mice. Two dimensions of tumor diameter were measured with calipers to calculate tumor volume (V=[(D + d)/2]^3^), where D and d represent larger and smaller diameters, respectively. Once the width of tumors reached 3-6 mm, 15 animals were randomly divided into three groups: control (normal saline), cisplatin (2 mg/kg, every other day), and cardamonin (20 mg/kg, every day). At 13 days after drug treatment, mice were sacrificed and tumors isolated.

### Statistical analysis

Our experiments and statistical analyses complied with the recommendations on experimental design and analysis in pharmacology. All data are presented as means ± SD. The statistical analysis was performed with using Student's *t*-test in GraphPad Prism. *P* values < 0.05 was considered as statistically significant.
